# Description of Eschar-Associated Rickettsial Diseases Using Passive Surveillance Data — United States, 2010–2016

**DOI:** 10.15585/mmwr.mm685152a2

**Published:** 2020-01-03

**Authors:** Naomi Drexler, Kristen Nichols Heitman, Cara Cherry

**Affiliations:** 1 Division of Vector-Borne Diseases, National Center for Emerging and Zoonotic Infectious Diseases, CDC.

Rickettsial eschars are necrotic lesions that occur at the site of tick or mite bites and represent locations of primary inoculation of spotted fever group *Rickettsia* and *Orientia* species. In the United States, eschars are hallmarks of less severe spotted fever diseases, including those caused by endemic agents such as *Rickettsia* *parkeri* (*1*) and *Rickettsia* species 364D (*2*), as well as several imported agents, including *Rickettsia africae, Rickettsia* *conorii,* and *Orientia tsutsugamushi*. Eschars generally do not occur with Rocky Mountain spotted fever (RMSF), a potentially deadly disease caused by *Rickettsia rickettsii* and have not been associated with *Ehrlichia* or *Anaplasma* species. The presence of eschars can help differentiate less severe spotted fever rickettsioses from RMSF and clarify the potential contributions of each within surveillance data. The lone star tick (*Amblyomma americanum),* the Gulf Coast tick (*Amblyomma maculatum*), and the Pacific Coast tick *(Dermacentor occidentalis)* are the three most common species of tick vectors that spread eschar-associated pathogens in the United States (*1*–*4*). Lone star and Gulf Coast ticks are vectors of *R. parkeri*, and Pacific Coast ticks are vectors of *Rickettsia* species 364D. In commonly available serologic assays, spotted fever group *Rickettsia* antigens cross-react, which presents a challenge when differentiating RMSF from other spotted fever rickettsioses. Incidence of spotted fever rickettsiosis continues to rise, with few cases providing species-specific laboratory evidence; therefore, the proportion of spotted fever rickettsioses caused by *R. rickettsii* remains unclear (*5*). This analysis serves as the first summary of eschar-associated rickettsial disease surveillance data in the United States. During 2010–2016, the presence or absence of eschars was reported in <20% of tickborne rickettsial disease (TBRD) cases. Eschar-associated illnesses represented a small percentage (1.1%) of TBRD cases. Among the 484 reported eschar-associated cases, 97 (20%) were classified as ehrlichiosis or anaplasmosis. Further research is needed to determine whether eschars associated with ehrlichiosis or anaplasmosis reflect a reporting error, a new finding, or the result of coinfection with another eschar-associated rickettsial pathogen.

Notifiable rickettsial diseases are reported to CDC through the National Notifiable Diseases Surveillance System, which also collects basic demographic information. Supplementary information is recorded through submission of TBRD supplemental case report forms, or extractions from state surveillance systems, and includes clinical details, diagnostic criteria, and patient outcomes. Since 2010, the CDC supplemental case report form* has requested information on eschars.

For this report, supplementary surveillance data collected by state and local health departments for illness with onset during 2010–2016 that were received and entered by CDC as of November 13, 2018, were summarized. TBRDs are not reportable conditions in Alaska and Hawaii, so no data from these states were included in this report. Case classifications were made according to the Council of State and Territorial Epidemiologists definitions (*6*,*7*). Confirmed cases were clinically compatible and had confirmatory diagnostic evidence obtained by seroconversion (fourfold change) in anti-*Ehrlichia, -Anaplasma,* or -*Rickettsia* immunoglobulin (Ig)G antibody titers by indirect immunofluorescence antibody assay or tested positive by polymerase chain reaction (PCR), immunohistochemistry, or culture. Probable cases were clinically compatible and included supportive laboratory evidence from serologic assays (including IgG- or IgM-positive antibodies reactive to *Ehrlichia, Anaplasma*, or *Rickettsia* species using immunofluorescence antibody assay or other serologic methods) or reported the presence of morulae (intracellular inclusion bodies in leukocytes) (*7*). Data were analyzed using SAS software (version 9.4; SAS Institute).

A rickettsial eschar begins as a small, painless papule that appears within a few days after the bite of an infected vector. The papule grows, becomes vesicular or pustular, and ulcerates forming a brown-to-black crust surrounded by a red annular halo (Figure 1). During 2010–2016, a total of 44,099 cases of TBRD with supplemental case report forms were reported to CDC, including 484 (1.1%) reported as eschar-associated TBRD; however, most case reports (35,749, 81.1%) were missing information on eschars altogether. Among reported eschar-associated cases, 387 (80.0%) were classified as spotted fever rickettsioses, 64 (13.2%) as *Ehrlichia chaffeensis* ehrlichiosis, 30 (6.2%) as *Anaplasma phagocytophilum* anaplasmosis, one (0.2%) as *Ehrlichia ewingii* ehrlichiosis, and two (0.4%) as undetermined ehrlichiosis/anaplasmosis. Notation of suspected spotted fever species is not required but was listed for 16 (4.1%) cases, including *R. africae* (11 cases), *R. parkeri* (two) and *R. conorii* (one), *Rickettsia* species 364D (one), and *Rickettsia akari* (one). No eschar-associated cases were associated with *R. rickettsii*.

**FIGURE 1:**
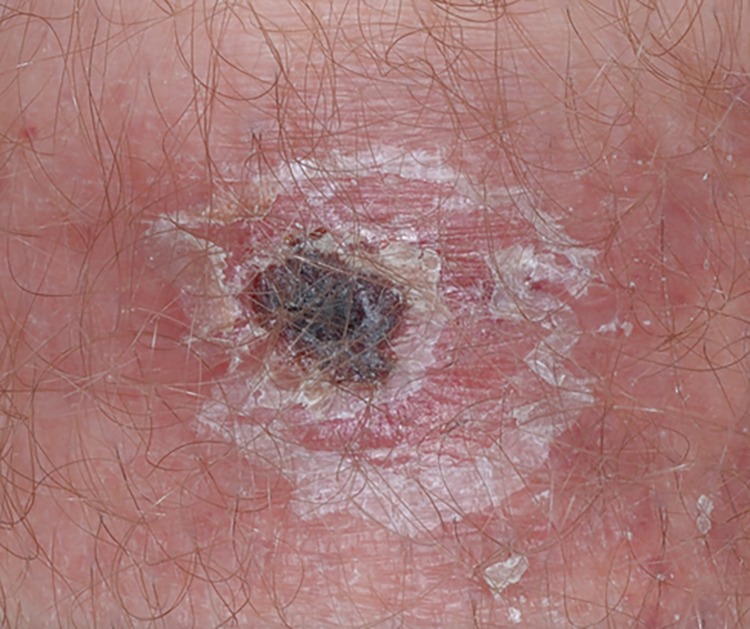
Rickettsial disease eschar from a patient with *Rickettsia parkeri* rickettsiosis Photo/CDC

Patients reporting eschar-associated illnesses were predominantly male (290, 59.9%), white (331, 68.4%), and non-Hispanic (402, 83.1%) (Table). Hospitalization (90, 18.6%) and death (1, 0.2%) occurred among a smaller proportion of patients with eschar-associated illness than among those with illness not associated with eschar (2,120, 27.0% and 21, 0.3%), respectively. Race and sex distributions were similar among patients with and without eschars. All but seven jurisdictions in which TBRD are reportable submitted information on the presence and absence of eschars during this period. Most eschar-associated cases (74.6%, 361) were reported from the South, compared with 60.3% (4,738) of cases not associated with eschar (Table). Most eschar-associated cases (462, 95.5%) were reported from states where ticks that transmit eschar-associated pathogens were present (Figure 2). A large proportion of all TBRD cases were missing travel history (30,455, 69.1%).

**TABLE:** Demographic characteristics and outcome indicators for tickborne rickettsial disease cases by eschar status — United States case report forms, 2010–2016

Characteristic	No. (%)			Chi-squared p-value*
	Eschar reported (n = 484)	No eschar reported (n = 7,866)	Missing information about eschars (n = 35,749)	
**Case classification**				p<0.001
Confirmed	42 (8.7)	1,093 (13.9)	11,145 (31.2)	
Probable	442 (91.3)	6,773 (86.1)	24,604 (68.8)	
**Sex**				p<0.001
Male	290 (59.9)	5,037 (64.0)	21,887 (61.2)	
Female	189 (39.0)	2,780 (35.3)	13,166 (36.8)	
Unknown	5 (1.0)	49 (0.6)	696 (2.0)	
**Race**				p<0.001
White	331 (68.4)	5,896 (75.0)	23,923 (66.9)	
Black	10 (2.1)	143 (1.8)	670 (1.9)	
American Indian/Alaska Native	8 (1.7)	40 (0.5)	776 (2.2)	
Asian/Pacific Islander	2 (0.4)	37 (0.5)	186 (0.5)	
Not specified/Unknown	133 (27.5)	1,750 (22.3)	10,194 (28.5)	
**Ethnicity**				p<0.001
Hispanic	10 (2.1)	203 (2.6)	677 (1.9)	
Non-Hispanic	402 (83.1)	6,267 (79.7)	21,668 (60.6)	
Unknown	72 (14.9)	1,396 (17.8)	13,404 (37.5)	
**Age group (yrs)**				p<0.001
<10	11 (2.3)	183 (2.5)	789 (3.1)	
10–19	14 (2.9)	387 (5.3)	1,350 (5.3)	
20–29	37 (7.6)	546 (7.4)	1,705 (6.7)	
30–39	45 (9.3)	827 (11.2)	2,408 (9.5)	
40–49	71 (14.7)	1,128 (15.3)	3,448 (13.5)	
50–59	99 (20.5)	1,547 (21.0)	5,269 (20.6)	
60–69	91 (18.8)	1,526 (20.7)	5,569 (21.9)	
≥70	79 (16.3)	1,220 (16.6)	4,960 (19.5)	
Unknown	37 (7.6)	502 (6.4)	10,261 (28.7)	
**U.S. Census region of residence^†^**				p<0.001
Northeast	24 (5.0)	608 (7.7)	10,576 (29.7)	
Midwest	71 (14.7)	2,385 (30.4)	11,881 (33.3)	
South	361 (74.6)	4,738 (60.3)	12,888 (36.1)	
West	28 (5.8)	125 (1.6)	329 (0.9)	
**Travel**				p<0.001
Yes	110 (22.7)	1,403 (17.8)	3,730 (10.4)	
No	162 (33.5)	1,678 (21.3)	6,562 (18.4)	
Unknown	212 (43.8)	4,785 (60.8)	25,457 (71.2)	
**Immunosuppressive condition**				p<0.001
Yes	62 (12.8)	765 (9.7)	2,109 (5.9)	
No	318 (65.7)	5,349 (68.0)	14,474 (40.5)	
Unknown	104 (21.5)	1,752 (22.3)	19,166 (53.6)	
**Hospitalization status**				p<0.001
Hospitalized	90 (18.6)	2,120 (27.0)	9,104 (25.5)	
Not hospitalized	368 (76.0)	5,559 (70.7)	17,529 (49.0)	
Unknown	26 (5.4)	187 (2.4)	9,116 (25.5)	
**Outcome**				p<0.001
Died	1 (0.2)	21 (0.3)	124 (0.4)	
Survived	434 (89.7)	7,351 (93.5)	26,895 (75.2)	
Unknown	49 (10.1)	494 (6.3)	8,730 (24.4)	

* Statistically significant difference (p<0.05) between eschar reporting categories using Chi-squared analysis.

^†^ *Northeast:* Connecticut, Maine, Massachusetts, New Hampshire, New Jersey, New York, Pennsylvania, Rhode Island, and Vermont. *Midwest:* Illinois, Indiana, Iowa, Kansas, Michigan, Minnesota, Missouri, Nebraska, North Dakota, Ohio, South Dakota, and Wisconsin. *South:* Alabama, Arkansas, Delaware, District of Columbia, Florida, Georgia, Kentucky, Louisiana, Maryland, Mississippi, North Carolina, Oklahoma, South Carolina, Tennessee, Texas, Virginia, and West Virginia. *West:* Alaska, Arizona, California, Colorado, Hawaii, Idaho, Montana, Nevada, New Mexico, Oregon, Utah, Washington, and Wyoming.

**FIGURE 2:**
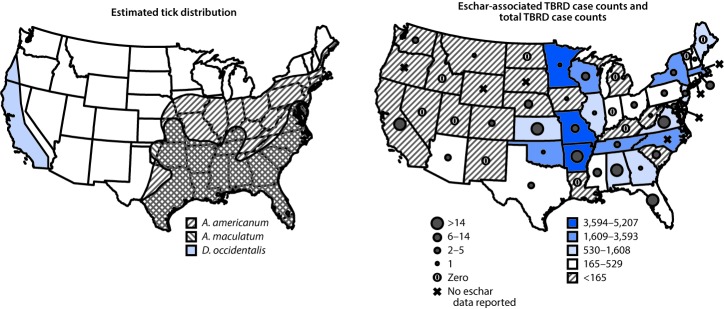
Estimated geographic range of Amblyomma americanum, Amblyomma maculatum,* and *Dermacentor occidentalis*^†^ and number of eschar-associated illnesses, compared with total reported tickborne rickettsial diseases (TBRDs)^§^ — United States, 2010–2016 * https://www.cdc.gov/ticks/geographic_distribution.html. ^†^ Bishopp FC, Trembley HL. Distribution and hosts of certain North American ticks. J Parasitol 1945;31:1–54. ^§^ TBRDs are not reportable conditions in Alaska and Hawaii; therefore, these states were not included in this figure.

Only 42 (8.7%) of 484 eschar-associated cases were confirmed, compared with 1,093 (13.9%) TBRD cases not associated with eschar (Table). Thirty-four (7.0%) reported eschar-associated cases were tested by PCR, one report described visualization of morulae, and 447 (92.0%) cases met confirmed or supportive laboratory criteria using serologic evidence; techniques were not mutually exclusive.

## Discussion

The presence of an eschar can aid in the clinical and epidemiologic differentiation of less severe spotted fever rickettsioses (e.g., Pacific Coast tick fever [*Rickettsia* species 364D] and *R. parkeri* rickettsiosis) from the more severe RMSF (*8*). Complete reporting of eschars might help to explain the proportions of spotted fever rickettsioses that are caused by less pathogenic spotted fever group *Rickettsia* and those caused by *R. rickettsii*. In addition, rickettsial eschars serve as an important clinical specimen; rickettsial DNA can be extracted from eschar lesions obtained by punch biopsies, by removing a portion of the eschar scab, or by swabbing the ulcerated area (*9*). PCR testing of eschar swabs and scabs provides confirmatory testing without a more invasive biopsy, although the pathogen cannot be cultured and immunohistochemistry cannot be performed on eschar swabs or scabs. To assist agencies that request rickettsial disease testing, CDC provides instructions for collection and submission of eschar swab and skin biopsy specimens.^†^

Demographic characteristics of patients with eschar-associated TBRD were similar to those of patients for whom eschars were not reported. Eschar-associated cases reported during this period were less likely to be confirmed, and less severe (as indicated by lower hospitalization and case-fatality rates), than were cases for which eschars were not reported, consistent with previously published studies (*1*–*3*,*8*). Gulf Coast and lone star ticks transmit several eschar-causing pathogens (including *R. parkeri*) and are prevalent in the southern United States, where most eschar-associated cases were reported (*1*,*3*,*10*). Although incomplete data on travel history limits the ability to draw conclusions regarding the geographic distribution of eschar-associated illnesses, the predominance of reported cases in areas with compatible vectors is consistent with expected distributions of eschar-associated illnesses, including *R. parkeri* rickettsiosis. Among the 22 cases reported from areas without these tick vectors, six were imported cases of either African tick bite fever (*R. africae*) or Mediterranean spotted fever (*R. conorii*) from Africa, but seven patients reported no travel and were primarily reported as having cases of anaplasmosis. Further investigation is needed to understand the occurrence of locally acquired eschar-associated illnesses in areas without known competent vectors.

Although the presence and frequency of spotted fever rickettsiosis associated with eschars was not surprising, the number of reported ehrlichiosis and anaplasmosis cases associated with eschars was unexpected. Approximately 20% of TBRD cases reporting the presence of an eschar during 2010–2016 were associated with cases of ehrlichiosis and anaplasmosis. Eschars had not previously been reported with *Anaplasma* or *Ehrlichia* species infections. Eschar-associated ehrlichiosis or anaplasmosis might represent a newly described clinical finding; signal coinfection with a spotted fever group *Rickettsia* and *Anaplasma* or *Ehrlichia* species; or indicate a reporting error. Coinfections could result from concomitant transmission of two pathogens carried by the same tick or from the bite of two separate tick species. Several pathogens are known to cocirculate: lone star ticks are known to transmit *E. chaffeensis, E. ewingii, R. parkeri,* and *Rickettsia amblyommatis*; however, coinfection has not been documented in humans (*3*,*10*). Further clinical research is needed to understand the importance of these findings.

The findings in this report are subject to at least three limitations. First, reported data regarding eschars come from passive surveillance systems and might not be representative of the overall disease incidence. Second, eschar reporting as part of TBRD surveillance is a relatively new element, introduced in 2010; as such, eschars might not be well understood or reported. Finally, conclusions about the demographic and geographic profiles of eschar-associated illnesses might be limited by missing data.

More complete reporting of eschars in surveillance data will help track this clinical feature as a hallmark of rickettsial disease and help differentiate less severe rickettsial diseases from deadly RMSF. Correct identification and complete documentation of eschar-associated TBRD surveillance data can enhance understanding of the impact of spotted fever rickettsioses in the United States.

**Summary**

**What is already known about this topic?**

Eschars are a clinical sign used to differentiate less severe rickettsioses from potentially deadly Rocky Mountain spotted fever.

**What is added by this report?**

Eschars are infrequently reported in tickborne rickettsial disease (TBRD) surveillance data and represent an underutilized resource to aid in distinguishing the various spotted fever group *Rickettsia*. Although 1% of total TBRD case reports during 2010–2016 documented the presence of an eschar, 81% of cases lacked information on eschars altogether.

**What are the implications for public health practice?**

Systematic reporting of the presence or absence of eschars on the TBRD case report form can improve the quality of surveillance data and enhance understanding of the impact of spotted fever rickettsioses in the United States.

## Footnotes

* The TBRD case report form used for this review can be found at https://www.cdc.gov/ticks/pdf/2010_TBRD_case_report.pdf; however, a case definition change for spotted fever rickettsiosis will go into effect January 1, 2020, and a new case report form is forthcoming.

† https://www.cdc.gov/ncezid/dvbd/specimensub/rickettsial-shipping.html.
